# Effects of contemporary agricultural land cover on Colorado potato beetle genetic differentiation in the Columbia Basin and Central Sands

**DOI:** 10.1002/ece3.5489

**Published:** 2019-07-31

**Authors:** Michael S. Crossley, Silvia I. Rondon, Sean D. Schoville

**Affiliations:** ^1^ Department of Entomology University of Wisconsin‐Madison Madison WI USA; ^2^ Department of Crop & Soil Sciences, Hermiston Agricultural Research and Extension Center Oregon State University Hermiston OR USA

**Keywords:** agroecosystems, crop rotation, genetic differentiation, landscape genetics, pest management

## Abstract

Landscape structure, which can be manipulated in agricultural landscapes through crop rotation and modification of field edge habitats, can have important effects on connectivity among local populations of insects. Though crop rotation is known to influence the abundance of Colorado potato beetle (CPB; *Leptinotarsa decemlineata* Say) in potato (*Solanum tuberosum* L.) fields each year, whether crop rotation and intervening edge habitat also affect genetic variation among populations is unknown. We investigated the role of landscape configuration and composition in shaping patterns of genetic variation in CPB populations in the Columbia Basin of Oregon and Washington, and the Central Sands of Wisconsin, USA. We compared landscape structure and its potential suitability for dispersal, tested for effects of specific land cover types on genetic differentiation among CPB populations, and examined the relationship between crop rotation distances and genetic diversity. We found higher genetic differentiation between populations separated by low potato land cover, and lower genetic diversity in populations occupying areas with greater crop rotation distances. Importantly, these relationships were only observed in the Columbia Basin, and no other land cover types influenced CPB genetic variation. The lack of signal in Wisconsin may arise as a consequence of greater effective population size and less pronounced genetic drift. Our results suggest that the degree to which host plant land cover connectivity affects CPB genetic variation depends on population size and that power to detect landscape effects on genetic differentiation might be reduced in agricultural insect pest systems.

## 1 INTRODUCTION

Landscape structure can affect population connectivity and population size, therefore influencing the distribution of genetic variation among populations (Manel, Schwartz, Luikart, & Taberlet, 2003; Storfer et al., 2007). These effects might be especially strong in agroecosystems, where land cover changes substantially through time (Hurt, 2002). Humans have deliberately manipulated agricultural land cover for millennia through the practice of crop rotation (White, 1970). While crop rotation is known to affect soil chemistry and pest abundance (Brust & King, 1994; Bullock, 1992; Havlin, Kissel, Maddux, Claassen, & Long, 1990; Meisner & Rosenheim, 2014; Triberti, Nastri, & Baldoni, 2016; Venter, Jacobs, & Hawkins, 2016), less is known about its effects on pest genetic variation (Miller et al., 2007).

The spatial distribution of agricultural pest populations can be conceptualized with a metapopulation model (Slatkin, 1977) in which the amount and location of suitable habitat (host plants) changes over time, and only a fraction of pest individuals find and colonize host patches each generation. Under this framework, the founding pest population size at a given host patch depends on the effective distance between plant host patches, which can be increased via resistance of the intervening land cover to pest dispersal (McRae, 2006). Crop rotation and field edge habitats can increase the distance between host patches and can potentially increase landscape resistance to pest dispersal (Huseth, Frost, & Knuteson, 2012), exerting effects on pest genetic variation by reducing gene flow and effective population size (Pannell & Charlesworth, 2000).

**Figure 1:**
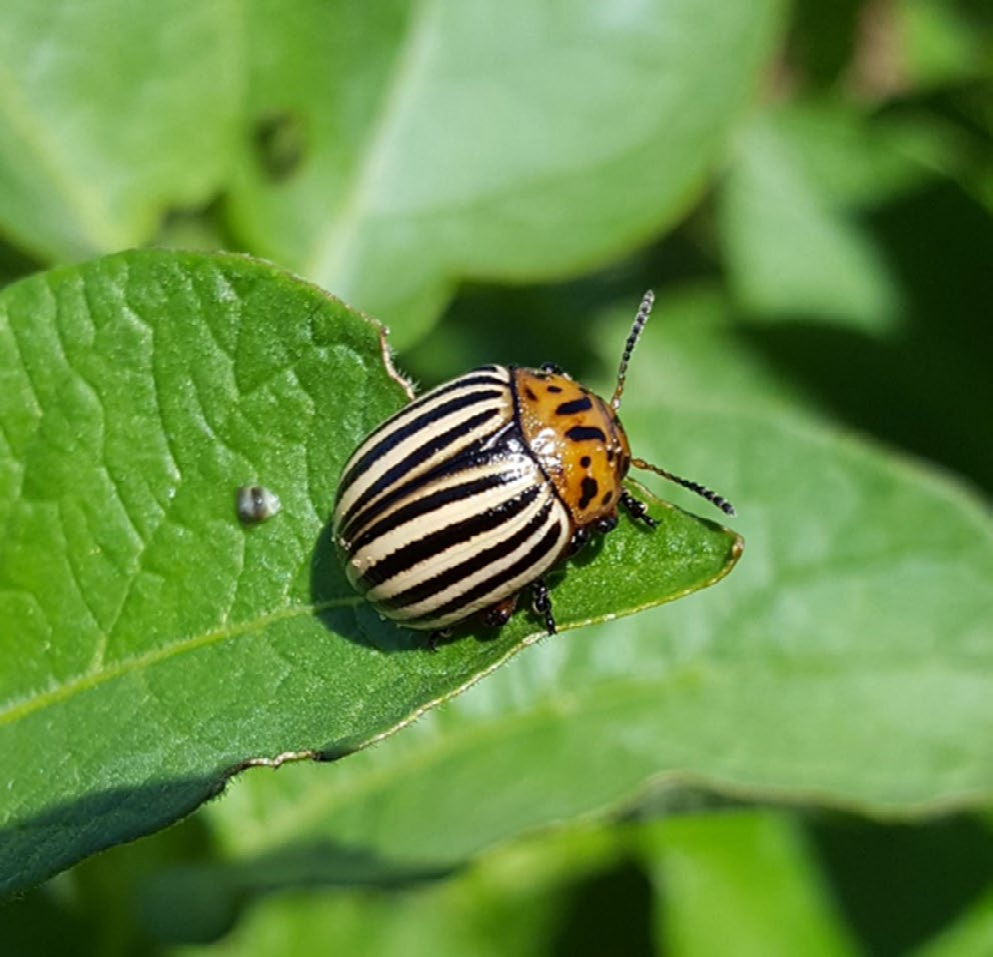
Adult Colorado potato beetle (*Leptinotarsa decemlineata* Say) feeding on potato (*Solanum tuberosum* L.) in a commercial potato field in Wisconsin, USA

One pest for which crop rotation has been central for management is the Colorado potato beetle (CPB; Figure 1), *Leptinotarsa decemlineata* Say, which is a major pest of potato (*Solanum tuberosum* L.) in North America, Europe, and Asia (Grapputo, Boman, & Lindström, 2005; Liu, Li, & Zhang, 2012; Tower, 1906; Walsh, 1866). In the United States, CPB exhibits a striking geographic pattern of insecticide resistance evolution, with rapid rates of evolution evident in the East, but slow rates of evolution in the West (Alyokhin et al., 2015; Crossley, Rondon, & Schoville, 2018; Olson, Dively, & Nelson, 2000; Szendrei, Grafius, Byrne, & Ziegler, 2012). Effects of agricultural landscape configuration (the distribution of different land cover types across the landscape) on CPB genetic variation might be an important contributor to this pattern of insecticide resistance evolution, via impacts on CPB gene flow and genetic diversity. However, it remains unclear how agricultural landscape structure affects CPB genetic variation and if land management practices are successful in reducing pest gene flow and genetic diversity.

Agricultural landscape structure can limit CPB dispersal primarily in the spring and fall, when beetles are searching for crops or solanaceous weeds and overwintering habitat. In the spring, beetles emerge from overwintering sites in the soil, and typically walk, rather than fly, in search of the nearest potato crop, orienting by olfactory cues (Boiteau, Alyokhin, & Ferro, 2003). Greater distances between potato fields together with unfavorable composition of the intervening landscape can impede navigation of CPB and make it difficult for CPB to reach a potato field; for example, water bodies, cereal crops, and grassland present barriers to CPB movement (Boiteau & MacKinley, 2015, 2017; Huseth et al., 2012; Lashomb & Ng, 1984). On the other hand, because potato attracts and retains CPB, areas with sparse potato land cover could unexpectedly enhance connectivity, because successful migrants must travel farther than beetles from areas with dense potato land cover. Though most CPB do not typically travel farther than 1 km to find potato in the spring (Sexson, & Wyman, 2005; Weisz, Smilowitz, & Fleischer, 1996), mass flights during warm spring days prior to potato emergence are not uncommon (M. S. Crossley, personal observations) and wind‐aided dispersal can facilitate migration over great distances (Hurst, 1975).

In the fall, beetles disperse to field margins, visually orienting toward the color contrasts created by wooded edges (Boiteau et al., 2003; Noronha & Cloutier, 1999; Weber & Ferro, 1993), though overwintering within potato fields can occur as well. If forested field margins act as a refuge for diapausing beetles, fall dispersal distances might be shorter in landscapes with a high proportion of forest. Overwintering habitat, the stepping stone between spring and fall dispersal, can also indirectly modulate beetle dispersal by decreasing winter survival. After beetles arrive at a field margin, they tunnel into the soil and enter diapause (Tower, 1906). Survival through the diapause state can be influenced by soil composition: sandy soils retain less moisture, and permit beetles to dig deeper—avoiding colder temperatures near the soil surface (Hiiesaar, Metspalu, Joudu, & Jogar, 2006; Weber & Ferro, 1993). Thus, dispersal might also be constrained by underlying soil texture.

In this study, we examined the relationship between landscape structure and patterns of genetic variation among CPB populations, addressing the questions: Does crop rotation affect patterns of genetic variation across growing regions? Do populations isolated by more non‐suitable habitat have greater genetic differentiation and less genetic diversity than other populations? We hypothesized that populations connected by more potato land cover, shorter rotation distances (in space), and more suitable overwintering habitat (forest land cover and sandy soils) would exhibit less genetic differentiation and higher genetic diversity, whereas populations separated by more grassland, grain crops, water bodies and greater rotational distances among potato fields would exhibit higher genetic differentiation and lower genetic diversity.

**Figure 2:**
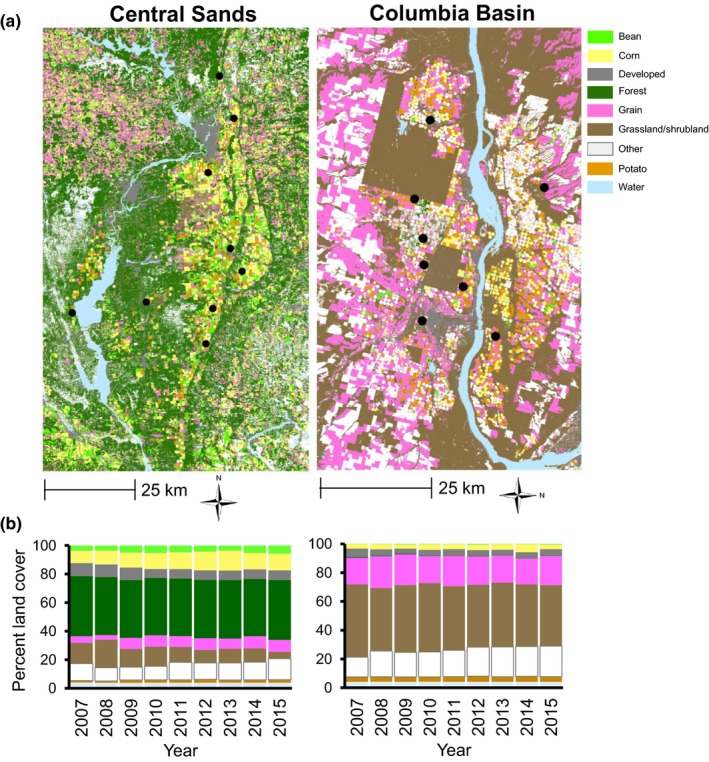
(a) Colorado potato beetle sample sites in 2014 and 2015, and land cover in 2015, reclassified to reflect the most abundant land cover types, in the Columbia Basin (Oregon and Washington) and Central Sands (Wisconsin). (b) Land cover proportions within each study extent from 2007 to 2015

We focused our study on regions representative of Northwestern and Midwestern potato agroecosystems: the Columbia Basin of Oregon and Washington, and the Central Sands of Wisconsin. CPB originally colonized the Central Sands during the 1860s (Riley, 1869; Walsh, 1866), but did not arrive in the Columbia Basin until the 1920s (Haegele & Wakeland, 1932; Mote, 1926). CPB population sizes tend to be smaller in the Columbia Basin than in the Central Sands (Crossley, Rondon, & Schoville, 2019). Landscape composition and climate also differ between these regions in important ways. The Central Sands is largely covered by forest, grassland (or pasture), and corn, while shrubland and wheat are the most abundant land cover types in the Columbia Basin (Figure 2). However, both landscapes share many less abundant agricultural land cover types (e.g., water, hay, and various specialty crops), including potato. The Columbia Basin experiences significantly less precipitation than the Central Sands (cumulative annual precipitation = 2,314 mm in the Columbia Basin vs. 5,820 mm in the Central Sands), a factor that could amplify any dispersal‐limiting effects of non‐suitable land cover in the Columbia Basin. Generally milder winters can also contribute to a higher proportion of “volunteer” potatoes (plants resulting from unharvested tubers remaining in the field from the previous year), which could act as an important bridge between overwintering and summer habitat.

## 2 MATERIALS AND METHODS

### 2.1 Beetle sampling and sequencing

We focused our sampling on a 12,855 km^2^ area in the Columbia Basin, and an 8,736 km^2^ area in the Central Sands, collecting CPB from commercial agricultural fields in eight locations in the Columbia Basin of Oregon and Washington and nine locations in the Central Sands of Wisconsin between 2014 and 2016 (Figure 2). From each location, we sampled 12 overwintered adult beetles from plants separated by at least three meters. Due to low abundance of CPB in commercial potato fields in the Columbia Basin, four sites represent samples from volunteer potatoes in nonhost crops. We collected beetles by hand and transported them in gallon jars for processing in the laboratory.

We obtained genetic data through a genotyping by sequencing (Elshire et al., 2011) protocol. The entire workflow, from DNA isolation to filtering of single nucleotide polymorphisms (SNPs), is described in Crossley, Chen, Groves, and Schoville (2017), Crossley et al. (2019). Illumina reads are available from the National Center for Biotechnology Information Short Read Archive (accession no. SRP098822 and PRJNA508767). The final SNP dataset consisted of 7,408 SNP loci for 93 beetles in the Columbia Basin and 106 beetles in the Central Sands and was used to detect associations between genetic differentiation and landscape resistance. In addition, we sequenced a 544 base‐pair fragment of the mitochondrial genome (COI‐COII), using the method described in Crossley et al. (2017), from 133 beetles in Oregon, and 50 beetles in Washington (and from several other sites in the USA; Tables S1 and S2), and combined these data with existing datasets (Crossley et al., 2017; Grapputo et al., 2005; Izzo, Chen, & Schoville, 2018). Mitochondrial DNA sequence data are available on GenBank (accession no. MK605454‐MK605457). The mitochondrial data provide an independent measure of population structure, and due to a smaller effective population size, can reveal strong patterns of genetic differentiation or changes in population size (Avise, Neigel, & Arnold, 1984). We visualized relationships among mitochondrial DNA haplotypes using a median joining network created in PopART (http://popart.otago.ac.nz/index.shtml; Bandelt, Forster, & Röhl, 1999), setting epsilon = 0.

### 2.2 Landscape composition and configuration

We obtained land cover rasters from the Cropland Data Layer (USDA‐NASS) for the years 2007 to 2015 and quantified the proportion of the landscape within our study extents that was occupied by major land cover types (which occupied at least 2% of the landscape in either study extent) each year using FRAGSTATS v4 (McGarigal, Cushman, & Ene, 2012). These land cover types were as follows: beans (Cropland Data Layer codes: 5, 42), corn (1, 12, 13), developed land (82, 121–124), fallow cropland (61), forest (63, 141–143), grains (21–39), grassland/shrubland (152, 176), potato (43), other (comprised every unspecified land cover type), water (83, 111), and wetlands (190, 195). We then generated binary rasters for each land cover type (target land cover = 1, all other land cover = 0) that accounted for more than one percent of each study extent using functions available in the “rgdal” (Bivand, Keitt, & Rowlingson, 2014) and “raster” (Hijmans, Etten, & Mattiuzzi, 2019) R packages in R 3.4.2 (R Core Team, 2017). We computed landscape resistance to dispersal (McRae, 2006) using the *commuteDistance()* function in the “gdistance” R package (van Etten, 2017). Resistance distances calculated with *commuteDistance()* and Circuitscape (McRae, Shah, & Edelman, 2013) have been shown to be equivalent (Marrotte & Bowman, 2017). We calculated geographic distance among sample sites using *pointDistance()* in the “gdistance” R package. Geographic distances were standardized by dividing by the standard deviation prior to landscape genetics analysis (with BEDASSLE; see below).

Prior to landscape genetics analysis, we removed variables that were highly correlated with geographic distance in both study extents, by examining Pearson correlation coefficients (using a threshold of *r* = .75) and axis loadings from principal components analysis (done with *prcomp* in R) on distances with all variables. This resulted in the removal of fallow cropland and wetlands. This analysis also revealed that “shrubland” and “grassland/pasture” classifications were frequently interchanged (possibly erroneously) among years; we therefore combined these land cover classifications into “grassland/shrubland” for landscape genetics analysis. We averaged resistance distances for each land cover type across years and standardized resistance and geographic distances by dividing by the standard deviation, as recommended for downstream landscape genetics analysis (Bradburd, Ralph, & Coop, 2013). We averaged across years because, though the locations of some land cover types changed among years between 2007 and 2015, the overall composition of the landscape was stable and constrained the extent of changes in configuration. Though we wanted to account for the small changes that we did observe among years, we did not test effects of specific years because the resolution of our genetic data was not appropriate for parsing effects of specific land cover types in specific years.

To assess the suitability of landscapes in the Columbia Basin and Central Sands for CPB overwintering, we quantified the percent sand in the soil, and the amount and configuration of potato field and forest edges. We calculated the percent sand in the soil from the Gridded Soil Survey Geographic Database (USDA‐NRCS, 2016) using the SSURGO OnDemand Dynamic Spatial Interpretations Tool (USDA‐NRCS, 2017). We calculated the edge and interspersion of potato and forest land cover using FRAGSTATS v4.2.1 (McGarigal et al., 2012) and compared the sensitivity of estimates at three scales: within 1, 5, and 10 km radii around samples sites. We first calculated the amount of potato and forest and the potato land cover *edge density* (the amount of edge per unit area; km/km^2^). We then calculated the potato land cover *interspersion and juxtaposition index*, which describes the degree of intermixing (as a percent) of potato with other land cover types; values of this index can range from 0 to 100, with high values indicating that potato land cover is adjacent to many other land cover types. Values of all metrics were represented as averages (± standard error) across sites and years (2007–2015).

### 2.3 Effect of landscape resistance on genetic differentiation

We estimated the effect of landscape resistance on genetic differentiation using the Bayesian Estimation of Differentiation in Alleles by Spatial Structure and Local Ecology framework (BEDASSLE; Bradburd et al., 2013). BEDASSLE employs a Bayesian approach to simultaneously identify the effect sizes of geographic distance and landscape resistance on differentiation in allele frequencies among populations. In addition to enabling a comparison of relative effect sizes (thus controlling for the effect of geographic distance while testing for an effect of landscape resistance), BEDASSLE does not assume a linear relationship between geographic distances, landscape resistance, and genetic differentiation (Bradburd et al., 2013). This is accomplished by estimating the rate of decay of allele frequency covariance with distance in the same model in which the effects sizes of geographic distance and landscape resistance are estimated. For each land cover variable, we ran the beta‐binomial model several (between five and ten) times and adjusted tuning parameters to achieve acceptance rates between 20% and 70% (Bradburd et al., 2013). When acceptance rates are less than 20%, we consider our search of parameter space to have been too narrow and inefficient (probably having settled on a local, rather than global, optimal set of parameter values). Conversely, when acceptance rates exceed 70%, we consider our search to have been too unconstrained and erratic to have precisely identified the optimal set of parameter values (Bradburd, 2014). We then ran 30 independent beta‐binomial Markov chains for four million steps per land cover variable. We assessed evidence for BEDASSLE model convergence by examining trace plots of the posterior probabilities and of the ratios of αE/αD (effect sizes of landscape resistance and geographic distance) and examining “scale reduction factors” calculated with a Rubin–Gelman test (using *gelman.diag()* in the “coda” R package (Plummer, Best, Cowles, & Vines, 2006)), which indicates model convergence when the variance in posterior probabilities within Markov chains is equivalent to the variance between Markov chains (upper 95% confidence interval of scale reduction factors approaches one). To test if effect sizes were sensitive to correlations among land cover types, we also jointly estimated effect sizes in a model including all land cover types.

### 2.4 Effect of crop rotation on genetic diversity

We expected that populations occupying areas with greater crop rotation distances (in space) would exhibit lower genetic diversity, due to the negative effect of crop rotation on CPB dispersal and abundance in potato fields (Sexson, & Wyman, 2005; Weisz et al., 1996). To test this, we first digitized potato fields that occurred the year prior to CPB sampling and within a 10 km radius of our sample sites, using the Cropland Data Layer (USDA‐NASS), Google Earth imagery, and ArcMap (ESRI). We chose a 10 km radius to conservatively account for potential long‐distance, wind‐aided dispersal events, though the maximum expected spring dispersal distance is only 1.5 km (Sexson, & Wyman, 2005; Weisz et al., 1996). We then calculated distances between sample sites and the centroids of potato fields, because the distance to the field centroid represents the average distance a CPB would have needed to disperse in order to colonize the sample site. We then calculated observed heterozygosity (*H_O_*) and average nucleotide diversity (π) in sampled CPB populations using the *populations* module of Stacks (Catchen, Amores, & Hohenlohe, 2011). Observed heterozygosity was the average among SNPs of the proportion of genotypes that were heterozygous. Nucleotide diversity was calculated as Weir and Cockeram's π, again averaged across SNPs for each population. Lastly, we regressed average nucleotide diversity and observed heterozygosity on the median crop rotation distance (the median taken from among all possible distances between the focal field in year *t* and all potato fields in year/*t* located within 10 km of the focal field) using *lm()* in R. We also calculated average pairwise *F*_ST_ *b*etween CPB populations using the method of Weir and Cockerham (1984) implemented with *calculate.all.pairwise.Fst()* in the “BEDASSLE” R package (Bradburd, 2014).

## 3 RESULTS

### 3.1 Genetic variation

Colorado potato beetle populations in the Columbia Basin exhibited lower genetic diversity and higher genetic differentiation than populations in the Central Sands. Nucleotide diversity (calculated from genotyping by sequencing data, including invariant sites) ranged from 0.0056 to 0.0063 in the Columbia Basin and 0.0073 and 0.0080 in the Central Sands. Observed heterozygosity was 0.51% ± 0.01% in the Columbia Basin and 0.67% ± 0.02% in the Central Sands. Considering only sites that were polymorphic among populations, observed heterozygosity was 19.4% ± 0.4% in the Columbia Basin and 21.6% ± 0.8% in the Central Sands. Pairwise *F*_ST_ was higher in the Columbia Basin (0.012 ± 0.001) than in the Central Sands (0.005 ± 0.000) and was highest between Columbia Basin and Central Sands populations (0.041 ± 0.000).

Mitochondrial DNA sequencing identified only two haplotypes in the Columbia Basin, one of which constituted 99% (182/183) of the Columbia Basin beetle samples, while the other was a singleton (Figure S1). Wisconsin, on the other hand, possessed seven haplotypes, three of which were unique to Wisconsin. The most common haplotype was shared between regions, reaching a frequency of 89% among Wisconsin beetle samples. Across all Northwestern and Midwestern beetle samples, haplotypes rarely differed by more than one nucleotide substitution (Figure S1).

### 3.2 Landscape structure

Land cover composition differed greatly between the Columbia Basin (Oregon and Washington) and the Central Sands (Wisconsin) study extents, with shrubland and grains (mainly wheat) dominating in the Columbia Basin, and forest, corn, and beans prevailing in the Central Sands between 2007 and 2015 (Figure 2). The percent land cover in potato was low in both regions (3.5% in the Columbia Basin; 1.8% in the Central Sands). There was on average more potato land cover between 2007 and 2015 in the Columbia Basin (22 km^2^) than in the Central Sands (16 km^2^), and the amount of potato land cover was more variable across years in the Central Sands (coefficient of variation: 19.3% vs. 5.8% in the Columbia Basin).

Factors that could affect overwintering success included the sandiness of the soil and the configuration of potato field and forest edges. We found that the proportion of sand in surface soils around sample sites was similarly high between regions (Figure S2), as expected for a crop cultivated in high‐drainage soils. The edge density of potato was greater surrounding Central Sands sample sites at 1 and 5 km scales, but not at 10 km (Table 1). The edge density of forest was also significantly greater surrounding Central Sands sites (Table 1), as expected based on regional differences in the abundance of forest land cover. Potato land cover was more interspersed among other land cover types in the Central Sands, regardless of scale (Table 1).

**Table 1:** Summary of class‐level landscape metrics for potato and forest land cover in the Columbia Basin (Oregon and Washington) and Central Sands (Wisconsin), averaged among years (2007–2015)

Scale (km)	Region	Potato				Forest
		Area (km^2^)	Total edge (km)	Edge density (km/km^2^)	Interspersion (%)	Edge density (km/km^2^)
1	Columbia Basin	0.4 (±0)	3.8 (±0.3)	1.2 (±0.1)	66.2 (±2.5)	8.7 (±1)
	Central Sands	0.6 (±0)	6.4 (±0.4)	2 (±0.1)	71.8 (±1.4)	38.9 (±3.4)
5	Columbia Basin	5 (±0.3)	51.2 (±2.8)	0.7 (±0)	67.5 (±1)	29 (±2.5)
	Central Sands	6 (±0.5)	72.6 (±5.5)	0.9 (±0.1)	80.2 (±0.9)	68.6 (±4.3)
10	Columbia Basin	19.7 (±0.8)	214.7 (±9.2)	0.7 (±0)	68.1 (±0.9)	4.1 (±3.3)
	Central Sands	17.8 (±1.6)	224.4 (±18.8)	0.7 (±0.1)	82.9 (±0.9)	77.6 (±3.1)

Note

Edge density is the length of land cover edge divided by the total length of edges of all land cover patches within the study extent. Interspersion describes the degree of intermixing of potato with other land cover types. N_Columbia Basin_ = 72 site x year combinations; N_Central Sands_ = 81.

Estimates of landscape resistance to gene flow (calculated from land cover rasters with *commuteDistance()* in R) for potato land cover were moderately correlated with estimates of landscape resistance for other land cover types in both regions. In the Columbia Basin, resistance distances for grain (abundance in landscape: 59%), forest (58%), and bean (57%) land cover exhibited the highest correlation with that of potato, whereas in the Central Sands, grassland/shrubland (abundance in landscape: 77%), corn (74%), water (69%), and sand (65%) exhibited the highest correlation.

### 3.3 Effects of landscape resistance on genetic differentiation

BEDASSLE models exhibited good convergence among chains after four million steps. Upper 95% confidence limits of scale reduction factors estimated with Gelman–Rubin tests were close to one (mean = 1.42; standard error = 0.07) (Table S3). Effect sizes of geographic distance and landscape resistance on allele frequency covariance were generally low, ranging from 10^–7^ to 9 and centered at 10^–3^ for geographic distance, and ranging from 10^–4^ to 4 and centering at 10^–3^ for landscape resistance. Values of *α2*, the parameter describing the rate of decay in allele frequency covariance with geographic distance, were significantly larger (covariance decayed more rapidly with increasing distance) in the Central Sands (Columbia Basin mean *α2* = 1.15, Central Sands mean *α2* = 1.58; *df* = 1, 8018; *F* = 1,017; *p *« .0001).

**Figure 3:**
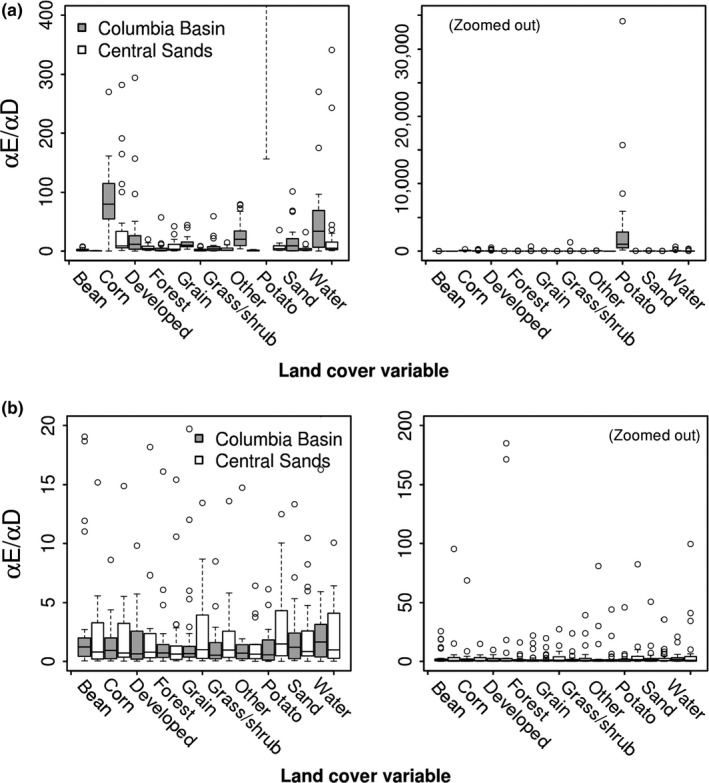
BEDASSLE estimates of the effect size of landscape resistance relative to geographic distance (*αE/αD*) on allele frequency differences in the Columbia Basin (Oregon and Washington) and the Central Sands (Wisconsin), with each land cover type analyzed separately (a) and all land cover types included in a single model (b). Boxplots represent the distribution of final parameter estimates across 30 independent Markov chains run for four million steps each. Right panels depict the same data as the left panels, but zoom out to visualize maximum outliers

Average relative effect sizes of contemporary land cover variables on genetic differentiation were consistently higher in the Columbia Basin (Figure 3, Table S3), being highest for potato (*αE*/*αD* = 3,329), followed by corn (87), water (67), and grassland/shrubland (50), but only the effect of potato in the Columbia Basin was significantly different from other land cover effects (*p* < .001). In the Central Sands, land cover types with relatively higher average effect sizes comprised corn (*αE*/*αD* = 40), forest (31), and water (29). When all land cover types were included in the same model, effect sizes became more even across land cover types (Figure 3, Table S3) and no land cover types exhibited statistically significant differences in average relative effect sizes. Overall reductions in effect size estimates were greater for the Columbia Basin than the Central Sands between models treating variables independently and together.

### 3.4 Relationships between crop rotation and genetic diversity

**Figure 4:**
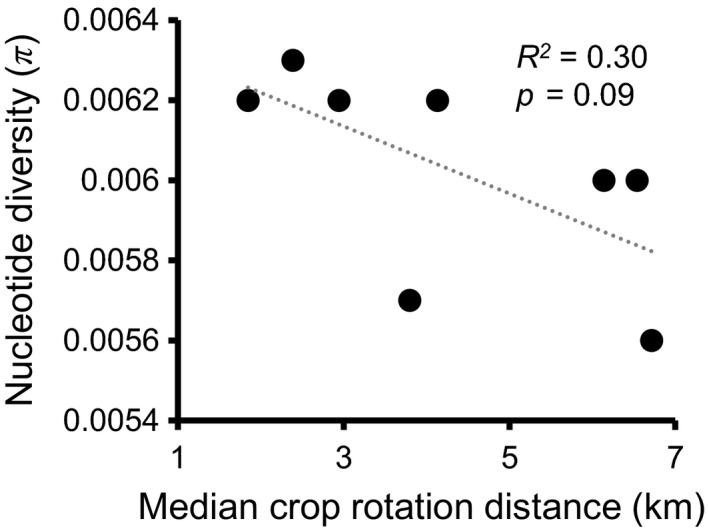
Relationship between genetic diversity (π) and median distance to potato fields within 10 km in the Columbia Basin. *P* and *R^2^* values were obtained through linear regression

Crop rotation distances around sample sites were generally shorter in the Central Sands (minimum: 0.7 ± 0.1 km; median: 3.8 ± 0.7 km) than in the Columbia Basin (minimum: 1.6 ± 0.4 km; median: 4.3 ± 0.7 km). Nucleotide diversity (π), but not observed heterozygosity, decreased with increasing crop rotation distance in the Columbia Basin, though this relationship was only marginally significant (*R* ^2^ = 30%; *p* = .09) (Figure 4). There was no relationship between crop rotation distance and genetic diversity in the Central Sands (Figure S3).

## 4 DISCUSSION

We used contemporary samples of land cover and SNP genotype data to detect associations between landscape resistance and genetic differentiation among CPB populations in the Columbia Basin (Oregon and Washington) and Central Sands (Wisconsin). We hypothesized that CPB genetic differentiation would decrease with increasing potato, shorter rotational distances (in space), and greater abundance of forest land cover and sandy soil between sites. Conversely, we expected genetic differentiation would increase with increasing grassland/shrubland, grain, water cover and with larger rotational distances. Models that independently considered land cover effects on genetic differentiation identified a strong effect of potato land cover in the Columbia Basin but no landscape effects in the Central Sands (Figure 3). Comparing land cover correlations and joint estimates of land cover effects in a single BEDASSLE model revealed no strong independent effects of any landscape variable (Figure 3, Table S3), suggesting agricultural land cover has weak, correlated effects on CPB genetic differentiation. Parsing effects of correlated land cover types on genetic differentiation is a significant challenge in landscape genetics (Cushman, McKelvey, Hayden, & Schwartz, 2006), one that may be especially difficult in agricultural landscapes, where land cover can turnover rapidly and is correlated in space and time.

Importantly, we also observed a correlation between potato field rotation distances and nucleotide diversity, suggesting that crop rotation, in addition to reducing the timing and abundance of CPB infestations in potato fields, also acts to reduce the genetic diversity of local CPB populations.

### 4.1 Land cover effects on genetic differentiation

In the Columbia Basin, sites connected by a high amount of potato land cover exhibited slightly lower genetic differentiation, consistent with the hypothesis that having an abundance of host plants facilitates dispersal and gene flow over the landscape. A similar relationship was found between genetic differentiation and grain (predominantly wheat) land cover. Wheat typically follows potato in crop rotation schemes in the Columbia Basin, but has not been a prevalent crop in the Central Sands since the early 1900s. Wheat is known to be a barrier to CPB dispersal by walking (Huseth et al., 2012; Lashomb & Ng, 1984; Schmera, Szentesi, & Jermy, 2007), but also harbors volunteer potatoes (plants growing from remnant, unharvested tubers) that serve as an early, systemic insecticide‐free food source (Xu & Long, 1997). Thus, the effect of wheat reducing genetic differentiation could be a consequence of its close spatiotemporal association with potato in Columbia Basin agroecosystems and suggests volunteer potatoes may be important facilitators of gene flow.

The lack of a clear association between landscape resistance and genetic differentiation in the Central Sands could mean that any effects of land cover on CPB gene flow were too weak to detect. Detection might be hindered if populations are very large (Wright, 1943) or are not in migration–drift equilibrium (Rousset, 1997): true effects of the landscape on gene flow might not be detectable given competing effects of genetic drift, migration, and selection in an evolutionarily young, frequently disturbed system. However, the effects observed in the Columbia Basin (Figure 2) suggest detection of some landscape effects are possible, though this may be a product of reduced effective population size in this region (as migration–drift equilibrium becomes re‐established more rapidly with smaller population sizes).

Instead, we suggest that gene flow among CPB populations in the Central Sands is relatively unconstrained by landscape composition or configuration and that this could be due to three factors. First, CPB population sizes are substantially higher in the Central Sands than in the Columbia Basin. CPB dispersal is density‐dependent (Boiteau et al., 2003; Harcourt, 1971), so higher population sizes could cause higher densities, facilitating higher gene flow as well as the maintenance of higher genetic diversity. Indeed, we frequently observe mass‐migration events in densely CPB‐populated Wisconsin potato fields. Second, land cover in the Central Sands is likely more suitable for overwintering success (there were higher amounts of potato and forest edge). Given that winter survivorship can be as low as 5% (Huseth & Groves, 2013), the amount of suitable overwintering habitat can have important impacts on successful migration between rotated potato fields. The realized contribution of landscape structure to overwintering success, however, will likely depend on interacting effects of climate and noncrop host plants. Lastly, alternative host plants could be enhancing landscape connectivity independently of potato land cover. We observe high densities of weedy *Solanum* species (*S. nitidibaccatum* Bitter and *S. carolinense* L.) in potato‐producing regions in the Midwest (M. S. Crossley, personal observations), whereas alternative host plants in the Columbia Basin tend to occur in isolated riparian habitats (Castillo Carrillo, Fu, Jensen, & Snyder, 2016). Incorporation of alternative host plant occurrence in agricultural landscapes could improve inferences about landscape connectivity for CPB populations.

### 4.2 Crop rotation and genetic diversity

Consistent with the absence of an effect of landscape configuration on CPB genetic differentiation among CPB populations in the Central Sands, we found no relationship between crop rotation distances and genetic diversity in the Central Sands. In contrast, we found decreasing genetic diversity with increasing crop rotation distance in the Columbia Basin. This regional difference could be due to the generally larger rotation distances observed around our sample sites in the Columbia Basin or to differences in the suitability of the landscape for CPB dispersal. Importantly, our analysis did not identify any land cover types that specifically impede gene flow, suggesting that effects of crop rotation on CPB genetic variation are related to the sensitivity of dispersing CPB to other environmental factors in the absence of host plant (potato) land cover. One such environmental factor could be climate: There is much lower moisture availability in the Columbia Basin than in the Central Sands, which could reduce the amount of time and distance over which CPB can disperse before succumbing to desiccation and starvation.

### 4.3 Management implications

Crop rotation is a powerful management practice, leveraging one of the most malleable features of the agricultural landscape: the spatiotemporal connectivity of crop land cover. The geographic distance between rotated potato fields and composition of the intervening landscape affects CPB dispersal and abundance (Huseth et al., 2012; Sexson, & Wyman, 2005). However, our data suggest that crop rotation does not always reduce gene flow and might have a limited effect on the spatial pattern of neutral and adaptive genetic variation in some landscapes. In regions like the Columbia Basin, however, crop rotation could have an important effect on patterns of genetic variation. The reduced genetic connectivity observed between CPB populations separated by low potato land cover suggests that increasing rotation distances (in space and time) could reduce rates of adaptive gene flow and levels of genetic diversity and could limit the long‐term viability of CPB populations in this region. Moving forward, we plan to investigate the importance of other environmental (e.g., climate, natural enemies) and operational (e.g., insecticide use) factors, in addition to landscape connectivity, in driving patterns of geographic variation in CPB genetic variation and adaptation to insecticides.

## CONFLICT OF INTEREST

The authors declare no competing interests.

## AUTHOR CONTRIBUTIONS

MSC conceived of the study, gathered and analyzed data, and wrote the manuscript. SIR gathered data, provided funding, and wrote the manuscript. SDS gathered data, provided funding, and wrote the manuscript.

## Data Availability

llumina reads are available from the National Center for Biotechnology Information Short Read Archive (accession no. SRP098822 and PRJNA508767). Mitochondrial DNA sequence data are available on GenBank (accession no. MK605454‐MK605457).
