# Supplementary material for: Effects of contemporary agricultural land cover on Colorado potato beetle genetic differentiation in the Columbia Basin and Central Sands
Source: Ecol Evol. 2019 Jul 31;9(16):9385–94. doi: 10.1002/ece3.5489 (PMC6706216; doi:10.1002/ece3.5489)
Supplement: Supplementary file 1 [file ECE3-9-9385-s001.docx]

**Supplementary Material for:**

Effects of contemporary agricultural land cover on Colorado potato beetle genetic differentiation in the Columbia Basin and Central Sands

**Supplementary Table 1**. Sample locations of Colorado potato beetles for mitochondrial DNA (544-bp fragment spanning COI-COII) sequencing done in this study. Haplotype IDs correspond to those presented in Supplementary Table 2.

| **State** | **Latitude** | **Longitude** | **Date collected** | **Haplotype ID** |
| --- | --- | --- | --- | --- |
| FL | 30.0827 | -83.033 | 4/30/2015 | H1 |
| FL | 30.0827 | -83.033 | 4/30/2015 | H1 |
| FL | 30.0827 | -83.033 | 4/30/2015 | H1 |
| FL | 30.0827 | -83.033 | 4/30/2015 | H1 |
| FL | 30.0827 | -83.033 | 4/30/2015 | H1 |
| FL | 30.0827 | -83.033 | 4/30/2015 | H1 |
| FL | 30.0827 | -83.033 | 4/30/2015 | H1 |
| FL | 30.0827 | -83.033 | 4/30/2015 | H1 |
| FL | 30.0827 | -83.033 | 4/30/2015 | H1 |
| FL | 30.0827 | -83.033 | 4/30/2015 | H1 |
| ID | 42.55025556 | -114.34259722 | 7/14/2014 | H1 |
| ID | 42.55025556 | -114.34259722 | 7/14/2014 | H1 |
| ID | 42.55025556 | -114.34259722 | 7/14/2014 | H1 |
| ID | 42.55025556 | -114.34259722 | 7/14/2014 | H1 |
| ID | 42.55025556 | -114.34259722 | 7/14/2014 | H1 |
| ID | 42.55025556 | -114.34259722 | 7/14/2014 | H1 |
| ID | 42.55025556 | -114.34259722 | 7/14/2014 | H1 |
| ID | 42.55025556 | -114.34259722 | 7/14/2014 | H1 |
| ID | 42.55025556 | -114.34259722 | 7/14/2014 | H1 |
| ID | 42.55025556 | -114.34259722 | 7/14/2014 | H1 |
| ID | 42.55025556 | -114.34259722 | 7/14/2014 | H1 |
| ID | 42.55025556 | -114.34259722 | 7/14/2014 | H1 |
| ID | 42.55025556 | -114.34259722 | 7/14/2014 | H1 |
| ID | 42.55025556 | -114.34259722 | 7/14/2014 | H1 |
| ID | 42.55025556 | -114.34259722 | 7/14/2014 | H1 |
| ID | 42.55025556 | -114.34259722 | 7/14/2014 | H1 |
| ID | 42.55025556 | -114.34259722 | 7/14/2014 | H1 |
| ID | 42.55025556 | -114.34259722 | 7/14/2014 | H1 |
| ID | 42.55025556 | -114.34259722 | 7/14/2014 | H1 |
| ID | 42.55025556 | -114.34259722 | 7/14/2014 | H1 |
| MD | 38.59 | -75.91 | 7/21/2015 | H1 |
| MD | 38.59 | -75.91 | 7/21/2015 | H1 |
| MD | 38.59 | -75.91 | 7/21/2015 | H1 |
| MD | 38.86 | -76.78 | 7/21/2015 | H1 |
| MD | 38.86 | -76.78 | 7/21/2015 | H1 |
| MD | 38.86 | -76.78 | 7/21/2015 | H1 |
| MD | 38.86 | -76.78 | 7/21/2015 | H1 |
| MD | 38.86 | -76.78 | 7/21/2015 | H1 |
| MD | 38.59 | -75.91 | 7/21/2015 | H1 |
| MD | 38.59 | -75.91 | 7/21/2015 | H1 |
| ME | 46.661319 | -68.020531 | 5/8/2015 | H1 |
| ME | 46.661319 | -68.020531 | 5/8/2015 | H1 |
| ME | 46.661319 | -68.020531 | 5/8/2015 | H1 |
| ME | 46.661319 | -68.020531 | 5/8/2015 | H1 |
| ME | 46.661319 | -68.020531 | 5/8/2015 | H1 |
| ME | 46.661319 | -68.020531 | 5/8/2015 | H1 |
| ME | 46.661319 | -68.020531 | 5/8/2015 | H1 |
| ME | 46.661319 | -68.020531 | 5/8/2015 | H1 |
| ME | 46.661319 | -68.020531 | 5/8/2015 | H1 |
| ME | 46.661319 | -68.020531 | 5/8/2015 | H1 |
| MI | 42.67598880 | -85.42935000 | 6/11/2014 | H1 |
| MI | 42.55487778 | -85.56504166 | 6/18/2014 | H1 |
| MI | 42.55487778 | -85.56504166 | 6/18/2014 | H1 |
| MI | 42.55487778 | -85.56504166 | 6/18/2014 | H1 |
| MI | 42.68034444 | -85.58897499 | 6/12/2014 | H1 |
| MI | 42.68034444 | -85.58897499 | 6/12/2014 | H1 |
| MI | 42.68034444 | -85.58897499 | 6/12/2014 | H1 |
| MI | 42.68034444 | -85.58897499 | 6/12/2014 | H1 |
| MI | 42.68034444 | -85.58897499 | 6/12/2014 | H1 |
| MI | 42.68034444 | -85.58897499 | 6/12/2014 | H1 |
| MI | 42.68034444 | -85.58897499 | 6/12/2014 | H1 |
| MI | 42.68034444 | -85.58897499 | 6/12/2014 | H1 |
| MI | 42.68034444 | -85.58897499 | 6/12/2014 | H1 |
| MI | 42.67598880 | -85.42935000 | 6/11/2014 | H1 |
| MI | 42.67598880 | -85.42935000 | 6/11/2014 | H1 |
| MI | 42.67598880 | -85.42935000 | 6/11/2014 | H1 |
| MI | 42.67598880 | -85.42935000 | 6/11/2014 | H1 |
| MI | 42.67598880 | -85.42935000 | 6/11/2014 | H1 |
| MI | 42.67598880 | -85.42935000 | 6/11/2014 | H1 |
| MI | 42.68034444 | -85.58897499 | 6/12/2014 | H1 |
| NJ | 39.7 | -75.29 | colony | H1 |
| NJ | 39.7 | -75.29 | colony | H1 |
| NJ | 39.7 | -75.29 | colony | H1 |
| NJ | 39.7 | -75.29 | colony | H1 |
| NJ | 39.7 | -75.29 | colony | H1 |
| NJ | 39.7 | -75.29 | colony | H1 |
| NJ | 39.7 | -75.29 | colony | H1 |
| NJ | 39.7 | -75.29 | colony | H1 |
| NJ | 39.7 | -75.29 | colony | H1 |
| NJ | 39.7 | -75.29 | colony | H1 |
| NY | 40.905657 | -72.752664 | 7/14/2015 | H1 |
| NY | 40.905657 | -72.752664 | 7/14/2015 | H1 |
| NY | 40.905657 | -72.752664 | 7/14/2015 | H1 |
| NY | 40.905657 | -72.752664 | 7/14/2015 | H1 |
| NY | 40.905657 | -72.752664 | 7/14/2015 | H1 |
| NY | 40.905657 | -72.752664 | 7/14/2015 | H1 |
| NY | 40.905657 | -72.752664 | 7/14/2015 | H1 |
| OR | 45.8549708 | -119.5325238 | 6/17/2015 | H1 |
| OR | 45.8549708 | -119.5325238 | 6/17/2015 | H1 |
| OR | 45.8549708 | -119.5325238 | 6/17/2015 | H1 |
| OR | 45.73573 | -119.84855 | 6/17/2015 | H1 |
| OR | 45.74437 | -119.61729 | 5/17/2016 | H1 |
| OR | 45.74437 | -119.61729 | 5/17/2016 | H1 |
| OR | 45.74437 | -119.61729 | 5/17/2016 | H1 |
| OR | 45.74437 | -119.61729 | 5/17/2016 | H1 |
| OR | 45.74437 | -119.61729 | 5/17/2016 | H1 |
| OR | 45.74437 | -119.61729 | 5/17/2016 | H1 |
| OR | 45.74437 | -119.61729 | 5/17/2016 | H1 |
| OR | 45.74437 | -119.61729 | 5/17/2016 | H1 |
| OR | 45.74437 | -119.61729 | 5/17/2016 | H1 |
| OR | 45.74437 | -119.61729 | 5/17/2016 | H1 |
| OR | 45.74437 | -119.61729 | 5/17/2016 | H1 |
| OR | 45.74437 | -119.61729 | 5/17/2016 | H1 |
| OR | 45.883 | -119.40623 | 5/16/2016 | H1 |
| OR | 45.883 | -119.40623 | 5/16/2016 | H1 |
| OR | 45.883 | -119.40623 | 5/16/2016 | H1 |
| OR | 45.883 | -119.40623 | 5/16/2016 | H1 |
| OR | 45.883 | -119.40623 | 5/16/2016 | H1 |
| OR | 45.883 | -119.40623 | 5/16/2016 | H1 |
| OR | 45.883 | -119.40623 | 5/16/2016 | H1 |
| OR | 45.883 | -119.40623 | 5/16/2016 | H1 |
| OR | 45.883 | -119.40623 | 5/16/2016 | H1 |
| OR | 45.883 | -119.40623 | 5/16/2016 | H1 |
| OR | 45.883 | -119.40623 | 5/16/2016 | H1 |
| OR | 45.883 | -119.40623 | 5/16/2016 | H1 |
| OR | 45.883 | -119.40623 | 5/16/2016 | H1 |
| OR | 45.883 | -119.40623 | 5/16/2016 | H1 |
| OR | 45.8980194 | -119.4376667 | 4/21/2015 | H1 |
| OR | 45.8980194 | -119.4376667 | 4/21/2015 | H1 |
| OR | 45.8980194 | -119.4376667 | 4/21/2015 | H1 |
| OR | 45.8980194 | -119.4376667 | 4/21/2015 | H1 |
| OR | 45.8980194 | -119.4376667 | 4/21/2015 | H1 |
| OR | 45.8980194 | -119.4376667 | 4/21/2015 | H1 |
| OR | 45.8980194 | -119.4376667 | 4/21/2015 | H1 |
| OR | 45.8980194 | -119.4376667 | 4/21/2015 | H1 |
| OR | 45.8980194 | -119.4376667 | 4/21/2015 | H1 |
| OR | 45.81934 | -119.28216 | 5/18/2016 | H1 |
| OR | 45.81934 | -119.28216 | 5/18/2016 | H1 |
| OR | 45.81934 | -119.28216 | 5/18/2016 | H1 |
| OR | 45.81934 | -119.28216 | 5/18/2016 | H1 |
| OR | 45.81934 | -119.28216 | 5/18/2016 | H1 |
| OR | 45.81934 | -119.28216 | 5/18/2016 | H1 |
| OR | 45.81934 | -119.28216 | 5/18/2016 | H1 |
| OR | 45.81934 | -119.28216 | 5/18/2016 | H1 |
| OR | 45.81934 | -119.28216 | 5/18/2016 | H1 |
| OR | 45.81934 | -119.28216 | 5/18/2016 | H1 |
| OR | 45.81934 | -119.28216 | 5/18/2016 | H1 |
| OR | 45.81934 | -119.28216 | 5/18/2016 | H1 |
| OR | 45.8206333 | -119.2845028 | 4/20/2015 | H1 |
| OR | 45.8206333 | -119.2845028 | 4/20/2015 | H1 |
| OR | 45.8206333 | -119.2845028 | 4/20/2015 | H1 |
| OR | 45.8206333 | -119.2845028 | 4/20/2015 | H1 |
| OR | 45.8206333 | -119.2845028 | 4/20/2015 | H1 |
| OR | 45.8206333 | -119.2845028 | 4/20/2015 | H1 |
| OR | 45.8206333 | -119.2845028 | 4/20/2015 | H1 |
| OR | 45.8206333 | -119.2845028 | 4/20/2015 | H1 |
| OR | 45.8206333 | -119.2845028 | 4/20/2015 | H1 |
| OR | 45.8206333 | -119.2845028 | 4/20/2015 | H1 |
| OR | 45.8206333 | -119.2845028 | 4/20/2015 | H1 |
| OR | 45.8206333 | -119.2845028 | 4/20/2015 | H1 |
| OR | 45.8206333 | -119.2845028 | 4/20/2015 | H1 |
| OR | 45.8206333 | -119.2845028 | 4/20/2015 | H1 |
| OR | 45.8206333 | -119.2845028 | 4/20/2015 | H1 |
| OR | 45.73573 | -119.84855 | 6/17/2016 | H1 |
| OR | 45.73573 | -119.84855 | 6/17/2016 | H1 |
| OR | 45.73573 | -119.84855 | 6/17/2016 | H1 |
| OR | 45.73573 | -119.84855 | 6/17/2016 | H1 |
| OR | 45.73573 | -119.84855 | 6/17/2016 | H1 |
| OR | 45.73573 | -119.84855 | 6/17/2016 | H1 |
| OR | 45.73573 | -119.84855 | 6/17/2016 | H1 |
| OR | 45.73573 | -119.84855 | 6/17/2016 | H1 |
| OR | 45.73573 | -119.84855 | 6/17/2016 | H1 |
| OR | 45.73573 | -119.84855 | 6/17/2016 | H1 |
| OR | 45.73573 | -119.84855 | 6/17/2016 | H1 |
| OR | 45.8549708 | -119.5325238 | 6/17/2015 | H1 |
| OR | 45.8549708 | -119.5325238 | 6/17/2015 | H1 |
| OR | 45.8549708 | -119.5325238 | 6/17/2015 | H1 |
| OR | 45.8549708 | -119.5325238 | 6/17/2015 | H1 |
| OR | 45.8549708 | -119.5325238 | 6/17/2015 | H1 |
| OR | 45.8549708 | -119.5325238 | 6/17/2015 | H1 |
| OR | 45.8549708 | -119.5325238 | 6/17/2015 | H1 |
| OR | 45.8549708 | -119.5325238 | 6/17/2015 | H1 |
| OR | 45.8549708 | -119.5325238 | 6/17/2015 | H1 |
| OR | 45.8549708 | -119.5325238 | 6/17/2015 | H1 |
| OR | 45.8549708 | -119.5325238 | 6/17/2015 | H1 |
| OR | 45.8549708 | -119.5325238 | 6/17/2015 | H1 |
| OR | 45.8549708 | -119.5325238 | 6/17/2015 | H1 |
| OR | 45.73573 | -119.84855 | 6/17/2015 | H1 |
| OR | 45.73573 | -119.84855 | 6/17/2015 | H1 |
| OR | 45.73573 | -119.84855 | 6/17/2015 | H1 |
| OR | 45.73573 | -119.84855 | 6/17/2015 | H1 |
| OR | 45.73573 | -119.84855 | 6/17/2015 | H1 |
| OR | 45.73573 | -119.84855 | 6/17/2015 | H1 |
| OR | 45.73573 | -119.84855 | 6/17/2015 | H1 |
| OR | 45.73573 | -119.84855 | 6/17/2015 | H1 |
| OR | 45.73573 | -119.84855 | 6/17/2015 | H1 |
| OR | 45.73573 | -119.84855 | 6/17/2015 | H1 |
| OR | 45.73573 | -119.84855 | 6/17/2015 | H1 |
| OR | 45.73573 | -119.84855 | 6/17/2015 | H1 |
| OR | 45.73573 | -119.84855 | 6/17/2015 | H1 |
| OR | 45.73573 | -119.84855 | 6/17/2015 | H1 |
| OR | 45.73573 | -119.84855 | 6/17/2015 | H1 |
| OR | 45.73573 | -119.84855 | 6/17/2015 | H1 |
| OR | 45.73573 | -119.84855 | 6/17/2015 | H1 |
| OR | 45.73573 | -119.84855 | 6/17/2015 | H1 |
| OR | 45.73573 | -119.84855 | 6/17/2015 | H1 |
| OR | 45.73573 | -119.84855 | 6/17/2015 | H1 |
| OR | 45.73573 | -119.84855 | 6/17/2015 | H1 |
| OR | 45.73573 | -119.84855 | 6/17/2015 | H1 |
| OR | 45.73573 | -119.84855 | 6/17/2015 | H1 |
| OR | 45.73573 | -119.84855 | 6/17/2015 | H1 |
| OR | 45.735755 | -119.86706 | 6/17/2015 | H1 |
| OR | 45.735755 | -119.86706 | 6/17/2015 | H1 |
| OR | 45.735755 | -119.86706 | 6/17/2015 | H1 |
| OR | 45.735755 | -119.86706 | 6/17/2015 | H1 |
| OR | 45.735755 | -119.86706 | 6/17/2015 | H1 |
| OR | 45.735755 | -119.86706 | 6/17/2015 | H1 |
| OR | 45.735755 | -119.86706 | 6/17/2015 | H1 |
| OR | 45.735755 | -119.86706 | 6/17/2015 | H1 |
| OR | 45.735755 | -119.86706 | 6/17/2015 | H1 |
| OR | 45.735755 | -119.86706 | 6/17/2015 | H1 |
| OR | 45.735755 | -119.86706 | 6/17/2015 | H1 |
| OR | 45.735755 | -119.86706 | 6/17/2015 | H1 |
| OR | 45.735755 | -119.86706 | 6/17/2015 | H1 |
| OR | 45.735755 | -119.86706 | 6/17/2015 | H1 |
| OR | 45.735755 | -119.86706 | 6/17/2015 | H1 |
| OR | 45.735755 | -119.86706 | 6/17/2015 | H1 |
| OR | 45.735755 | -119.86706 | 6/17/2015 | H1 |
| OR | 45.735755 | -119.86706 | 6/17/2015 | H1 |
| WA | 45.97081 | -119.28996 | 5/17/2016 | H1 |
| WA | 45.97081 | -119.28996 | 5/17/2016 | H1 |
| WA | 45.97081 | -119.28996 | 5/17/2016 | H1 |
| WA | 45.97081 | -119.28996 | 5/17/2016 | H1 |
| WA | 45.97081 | -119.28996 | 5/17/2016 | H1 |
| WA | 45.97081 | -119.28996 | 5/17/2016 | H1 |
| WA | 45.97081 | -119.28996 | 5/17/2016 | H1 |
| WA | 45.97081 | -119.28996 | 5/17/2016 | H1 |
| WA | 45.97081 | -119.28996 | 5/17/2016 | H1 |
| WA | 46.405872 | -119.1344298 | 6/17/2015 | H1 |
| WA | 46.405872 | -119.1344298 | 6/17/2015 | H1 |
| WA | 46.405872 | -119.1344298 | 6/17/2015 | H1 |
| WA | 46.405872 | -119.1344298 | 6/17/2015 | H1 |
| WA | 46.405872 | -119.1344298 | 6/17/2015 | H1 |
| WA | 46.405872 | -119.1344298 | 6/17/2015 | H1 |
| WA | 46.405872 | -119.1344298 | 6/17/2015 | H1 |
| WA | 46.405872 | -119.1344298 | 6/17/2015 | H1 |
| WA | 46.405872 | -119.1344298 | 6/17/2015 | H1 |
| WA | 46.405872 | -119.1344298 | 6/17/2015 | H1 |
| WA | 46.405872 | -119.1344298 | 6/17/2015 | H1 |
| WA | 46.25753889 | -119.18862500 | 6/16/2014 | H1 |
| WA | 46.25753889 | -119.18862500 | 6/16/2014 | H1 |
| WA | 46.25753889 | -119.18862500 | 6/16/2014 | H1 |
| WA | 46.25753889 | -119.18862500 | 6/16/2014 | H1 |
| WA | 46.25753889 | -119.18862500 | 6/16/2014 | H1 |
| WA | 46.25753889 | -119.18862500 | 6/16/2014 | H1 |
| WA | 46.25753889 | -119.18862500 | 6/16/2014 | H1 |
| WA | 46.25753889 | -119.18862500 | 6/16/2014 | H1 |
| WA | 46.25753889 | -119.18862500 | 6/16/2014 | H1 |
| WA | 46.25753889 | -119.18862500 | 6/16/2014 | H1 |
| WA | 46.25753889 | -119.18862500 | 6/16/2014 | H1 |
| WA | 46.25753889 | -119.18862500 | 6/16/2014 | H1 |
| WA | 46.25753889 | -119.18862500 | 6/16/2014 | H1 |
| WA | 46.25753889 | -119.18862500 | 6/16/2014 | H1 |
| WA | 46.25753889 | -119.18862500 | 6/16/2014 | H1 |
| WA | 46.25753889 | -119.18862500 | 6/16/2014 | H1 |
| WA | 46.25753889 | -119.18862500 | 6/16/2014 | H1 |
| WA | 46.25753889 | -119.18862500 | 6/16/2014 | H1 |
| WA | 46.25753889 | -119.18862500 | 6/16/2014 | H1 |
| WA | 46.25753889 | -119.18862500 | 6/16/2014 | H1 |
| WA | -119.74209 | 45.99272 | 5/18/2016 | H1 |
| WA | -119.74209 | 45.99272 | 5/18/2016 | H1 |
| WA | -119.74209 | 45.99272 | 5/18/2016 | H1 |
| WA | -119.74209 | 45.99272 | 5/18/2016 | H1 |
| WA | -119.74209 | 45.99272 | 5/18/2016 | H1 |
| WA | -119.74209 | 45.99272 | 5/18/2016 | H1 |
| WA | -119.74209 | 45.99272 | 5/18/2016 | H1 |
| WA | -119.74209 | 45.99272 | 5/18/2016 | H1 |
| WA | -119.74209 | 45.99272 | 5/18/2016 | H1 |
| WA | -119.74209 | 45.99272 | 5/18/2016 | H1 |
| WI | -89.459608 | 44.167753 | 6/3/2015 | H1 |
| WI | -89.459608 | 44.167753 | 6/3/2015 | H1 |
| WI | -89.459608 | 44.167753 | 6/3/2015 | H1 |
| WI | -89.459608 | 44.167753 | 6/3/2015 | H1 |
| WI | -89.459608 | 44.167753 | 6/3/2015 | H1 |
| WI | -89.459608 | 44.167753 | 6/3/2015 | H1 |
| WI | -89.459608 | 44.167753 | 6/3/2015 | H1 |
| WI | -89.459608 | 44.167753 | 6/3/2015 | H1 |
| WI | -89.459608 | 44.167753 | 6/3/2015 | H1 |
| WI | -89.459608 | 44.167753 | 6/3/2015 | H1 |
| WI | -89.459608 | 44.167753 | 6/3/2015 | H1 |
| WI | 43.70077500 | -90.68651660 | 7/2/2014 | H1 |
| WI | 43.70077500 | -90.68651660 | 7/2/2014 | H1 |
| WI | 43.70077500 | -90.68651660 | 7/2/2014 | H1 |
| WI | 43.70077500 | -90.68651660 | 7/2/2014 | H1 |
| WI | 43.70077500 | -90.68651660 | 7/2/2014 | H1 |
| WI | 43.70077500 | -90.68651660 | 7/2/2014 | H1 |
| WI | 43.70077500 | -90.68651660 | 7/2/2014 | H1 |
| WI | -89.459608 | 44.167753 | 6/3/2015 | H1 |
| MI | 42.55487778 | -85.56504166 | 6/18/2014 | H2 |
| MI | 42.55487778 | -85.56504166 | 6/18/2014 | H3 |
| MI | 42.55487778 | -85.56504166 | 6/18/2014 | H4 |
| MI | 42.55487778 | -85.56504166 | 6/18/2014 | H5 |
| OR | 45.73573 | -119.84855 | 6/17/2016 | H6 |

**Supplementary Table 2**. Colorado potato beetle mitochondrial DNA (544-bp fragment spanning COI-COII) haplotype composition of samples (grouped by state) sequenced in this study. FL=Florida; ID=Idaho; MD=Maryland; ME=Maine; MI=Michigan; NJ=New Jersey; NY=New York; OR=Oregon; WA=Washington; WI=Wisconsin. Specific locations were haplotypes H1-H6 were found are indicated in Supplementary Table 1.

| Haplotype | FL | ID | MD | ME | MI | NJ | NY | OR | WA | WI |
| --- | --- | --- | --- | --- | --- | --- | --- | --- | --- | --- |
| H1 | 10 | 20 | 10 | 10 | 19 | 10 | 7 | 132 | 50 | 19 |
| H2 | 0 | 0 | 0 | 0 | 1 | 0 | 0 | 0 | 0 | 0 |
| H3 | 0 | 0 | 0 | 0 | 1 | 0 | 0 | 0 | 0 | 0 |
| H4 | 0 | 0 | 0 | 0 | 1 | 0 | 0 | 0 | 0 | 0 |
| H5 | 0 | 0 | 0 | 0 | 2 | 0 | 0 | 0 | 0 | 0 |
| H6 | 0 | 0 | 0 | 0 | 0 | 0 | 0 | 1 | 0 | 0 |

**Supplementary Table 3**. Summary of effect sizes of landscape resistance for each land cover type relative to geographic distance (αE/αD) on allele frequency differences among CPB populations collected in the Columbia Basin (Oregon & Washington) and Central Sands (Wisconsin). Data are presented from models treating each landscape variable individually (“Individual”) and together (“All”). αE/αD values are presented as mean (standard error), summarizing results from 30 independent MCMC chains consisting of 40 million steps each. Scale Reduction Factors were calculated using Gelman-Rubin tests, and are presented as factor estimate (upper 95% confidence interval).

| **Region** | **Variable** | **αE/αD** | |  | **Scale Reduction Factor** | |
| --- | --- | --- | --- | --- | --- | --- |
|  |  | **Individual** | **All** |  | **Individual** | **All** |
| Columbia Basin | bean | 2.3 (0.4) | 4 (1.3) |  | 1.08 (1.13) | 1.03 (1.05) |
|  | corn | 86.7 (9.9) | 3.9 (2.5) |  | 1.67 (2.09) | 1.04 (1.06) |
|  | developed | 46.6 (19.7) | 9.6 (7.9) |  | 1.12 (1.19) | 1.02 (1.04) |
|  | forest | 4.9 (1.9) | 1.7 (0.6) |  | 1.04 (1.06) | 1.03 (1.05) |
|  | grain | 13 (1.8) | 2.3 (0.8) |  | 1.08 (1.13) | 1.02 (1.03) |
|  | grassland/shrubland | 50.2 (43.5) | 2.1 (0.9) |  | 1.23 (1.38) | 1.03 (1.05) |
|  | other | 28.3 (4.3) | 5.3 (3.1) |  | 1.12 (1.2) | 1.06 (1.09) |
|  | potato | 3329 (1211.2) | 2.8 (1.7) |  | 1.27 (1.46) | 1.05 (1.09) |
|  | sand | 17 (4.3) | 3.8 (1.9) |  | 1.47 (1.71) | 1.06 (1.1) |
|  | water | 67 (23.4) | 2.9 (0.9) |  | 1.48 (1.74) | 1.05 (1.08) |
| Central Sands | bean | 0.5 (0.1) | 5.1 (3.3) |  | 1.41 (1.68) | 1.03 (1.04) |
|  | corn | 40.4 (13) | 34.6 (32.6) |  | 1.16 (1.25) | 1.03 (1.04) |
|  | developed | 6 (0.9) | 14 (8.5) |  | 1.6 (1.93) | 1.03 (1.05) |
|  | forest | 30.9 (23.6) | 27.2 (24.8) |  | 1.35 (1.55) | 1.03 (1.05) |
|  | grain | 2.3 (0.5) | 20.5 (17.4) |  | 1.18 (1.3) | 1.02 (1.03) |
|  | grassland/shrubland | 3.4 (0.6) | 3.8 (1.6) |  | 1.02 (1.04) | 1.03 (1.05) |
|  | other | 1.1 (0.1) | 14.3 (11.1) |  | 1.24 (1.38) | 1.05 (1.07) |
|  | potato | 6.5 (1.2) | 5.6 (2.8) |  | 1.1 (1.16) | 1.05 (1.09) |
|  | sand | 4 (1.1) | 3.2 (1.3) |  | 1.53 (1.88) | 1.06 (1.09) |
|  | water | 28.9 (13.4) | 7.7 (3.7) |  | 1.06 (1.1) | 1.04 (1.06) |

**
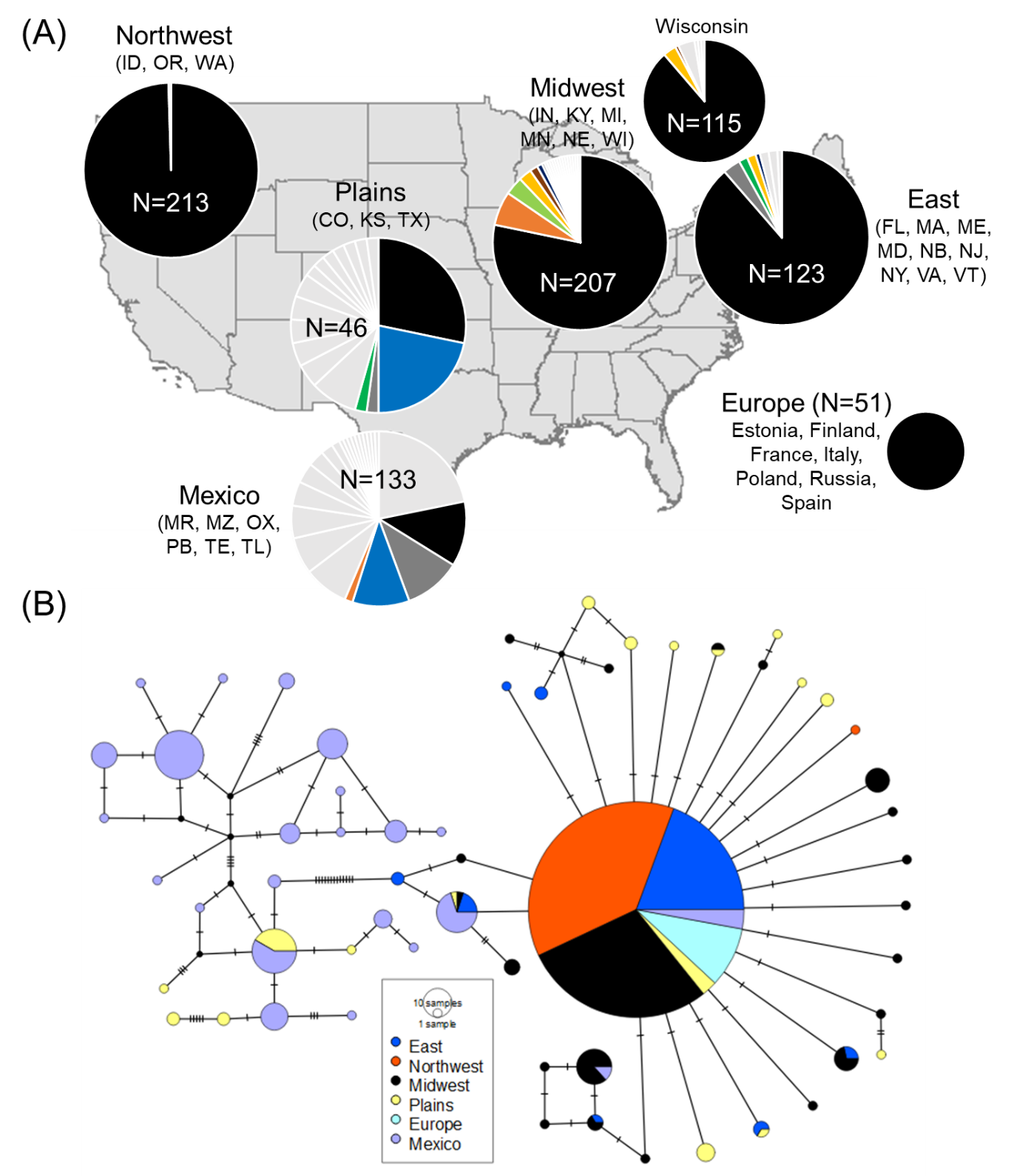
**

**Supplementary Figure 1**. (A) Colorado potato beetle mitochondrial DNA (544-bp fragment spanning COI-COII) haplotype frequencies among regions in North America, Mexico, and Europe. Data were combined from this study (293 beetle sequences) and Izzo et al. (2018; 227 sequences), Crossley et al. (2017; 144 sequences), and Grapputo et al. (2005; 109 sequences). (B) Median joining network depicting relationships among mitochondrial DNA haplotypes in Central and North America and Europe. Nodes correspond to haplotypes, node size represents the number of individual beetles with that haplotype, and hash marks on lines represent single base-pair differences between connected haplotypes.


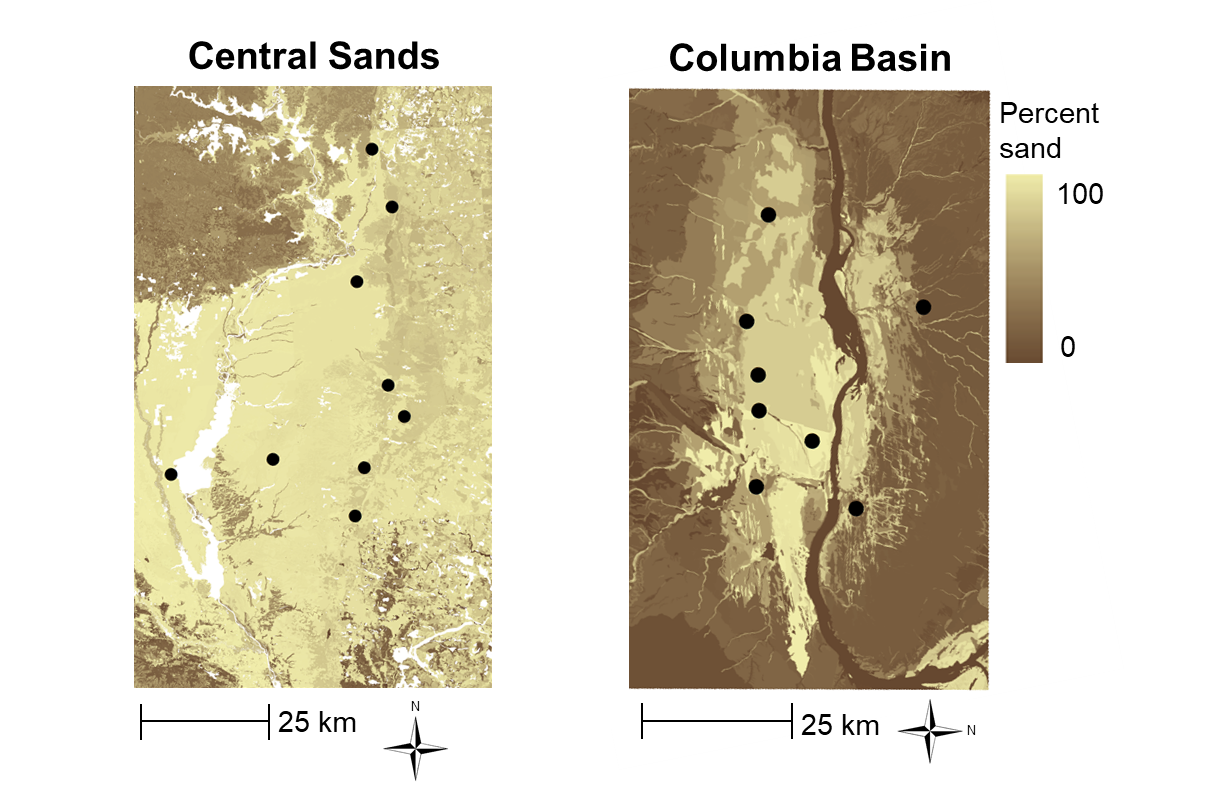


**Supplementary Figure 2**. Maps of proportion sand in surface soils in the Columbia Basin and Central Sands.

**
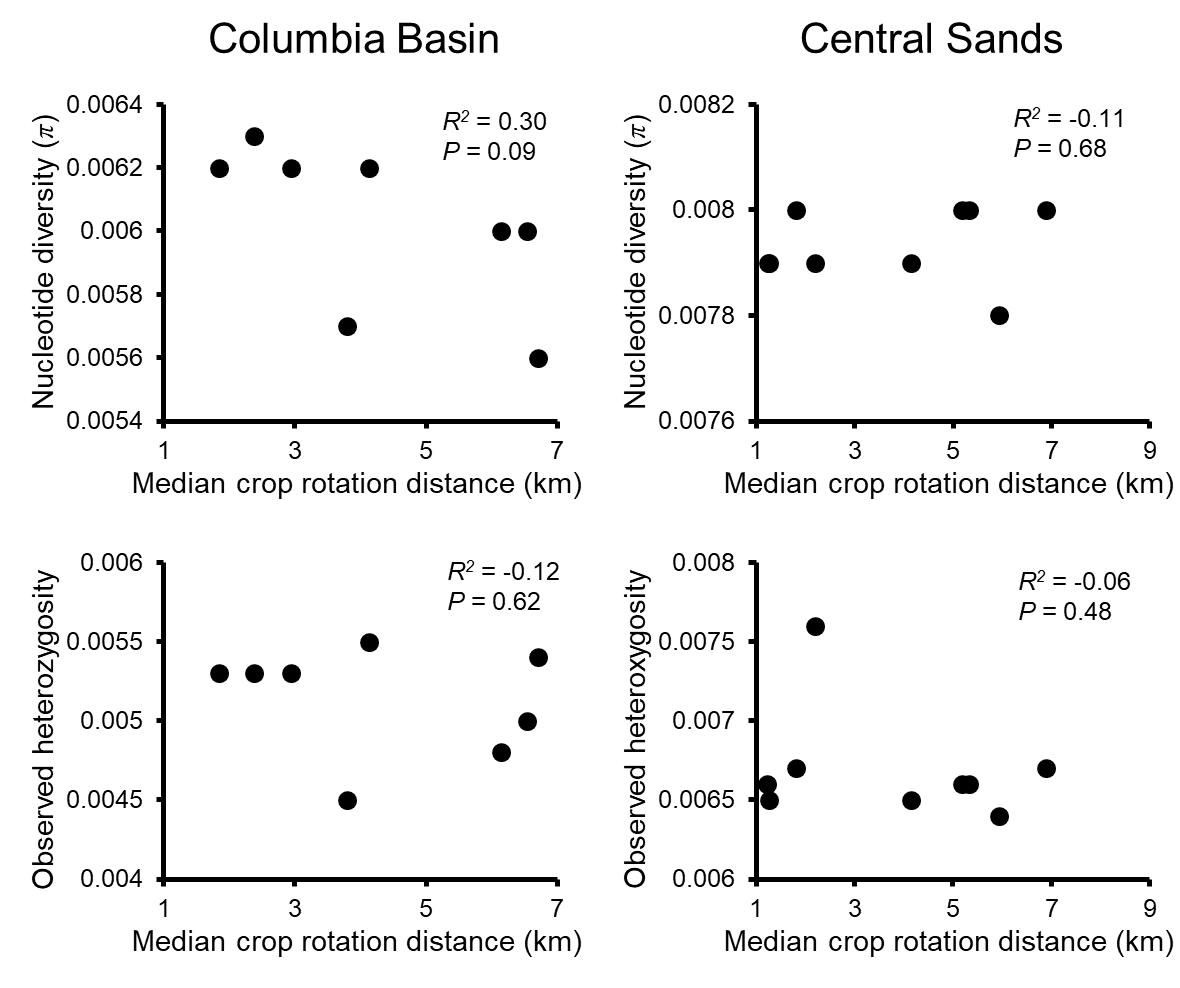
**

**Supplementary Figure 3**. Relationship between genetic diversity (nucleotide diversity and observed heterozygosity) and median distance to the previous year’s potato fields, limited to those within 10 km. *P* and *R^2^* values were obtained through linear regression.
